# The significance of chronic kidney disease, heart failure and cardiovascular disease for mortality in type 1 diabetes: nationwide observational study

**DOI:** 10.1038/s41598-022-22932-4

**Published:** 2022-10-26

**Authors:** Björn Eliasson, Lovisa Lyngfelt, Sven-Olof Strömblad, Stefan Franzén, Katarina Eeg-Olofsson

**Affiliations:** 1 Department of Medicine, Sahlgrenska University Hospital, 413 45 Gothenburg, Sweden; 2 National Diabetes Register, Centre of Registries in Region Western Sweden, Gothenburg, Sweden; 3 Institute of Medicine, University of Gothenburg, Sahlgrenska University Hospital, Gothenburg, Sweden; 4 Primary Care Global Business Unit, Sanofi, Stockholm, Sweden; 5 Health Metrics, School of Public Health and Community Medicine, University of Gothenburg, Gothenburg, Sweden

**Keywords:** Type 1 diabetes, Epidemiology

## Abstract

People with type 1 diabetes have a substantially increased risk of premature death. This nationwide, register-based cohort study evaluated the significance of risk factors and previous cardiovascular disease (CVD), heart failure and chronic kidney disease (CKD), for mortality in type 1 diabetes. Nationwide, longitudinal, register-based cohort study. Patients (n = 36,303) listed in the Swedish National Diabetes Register between January 1 2015 and December 31 2017 were included and followed until December 31, 2018. Data were retrieved from national health registries through each patient's unique identifier, to capture data on clinical characteristics, outcomes, or deaths, to describe mortality rates in risk groups. The mean follow-up time was 3.3 years, with 119,800 patient years of observation and 1127 deaths, corresponding to a crude overall mortality of 0.92% deaths/year. Statistically significant increased risk in multivariate analyzes was found in older age groups, in men, and in underweight or people with normal BMI, high HbA1c or blood pressure. A history of CVD, albuminuria and advanced stages of CKD was associated with an increased risk of mortality. Each combination of these conditions further increased the risk of mortality. These results emphasize the importance of risk factors and cardiovascular and renal diabetes complications. People with a combination of CKD, CVD, and heart failure, exhibit a markedly increased risk of dying prematurely. These findings provide strong arguments for optimized and individualized treatment of these groups of people with type 1 diabetes in clinical everyday life.

## Introduction

People with type 1 diabetes have a substantially increased risk of premature death and shorter life expectancy compared to the general population, with cardiovascular disease (CVD) being the leading cause of excess mortality^1,2^. Low age at onset of type 1 diabetes appears to be a particularly important determinant of cardiovascular outcomes, as well as of survival^3^.

Early cardiovascular mortality in diabetes has generally been attributed to classic cardiovascular risk factors, including overweight and obesity, elevated plasma glucose and blood lipid levels, and hypertension. Unlike the case of type 2 diabetes, however, people with type 1 diabetes are believed to be even more affected by elevated glucose levels in the long term, with vascular damage and premature death as results^3–5^. Hyperglycemia thus leads to microvascular complications, such as diabetic kidney disease, and is strongly associated with various manifestations of cardiovascular disease as well as heart failure^6,7^.

In the cardiorenal syndrome, dysfunction of either the kidneys or the heart has been suggested to lead to impaired function of the other, resulting in a synergistic, detrimental effect^8,9^. Hyperglycemia is thought to be a crucial underlying factor and several observational studies have shown associations between both HbA1c and disease duration, and development of cardiorenal complications and death^10–12^. Observational studies have shown that the trend for excess over-all mortality is declining, but the incidence of type 1 diabetes is still increasing^1,13–15^. The incidence of chronic kidney disease (CKD) in type 1 diabetes has also been suggested to be declining in recent decades, which could possibly be due to improved methods for optimizing glycemic control and antihypertensive treatment^16,17^.

The current study aims to expand and update the results of previous reports on cardiovascular over-risk and mortality for type 1 diabetes, and to describe the significance of risk factors and co-morbidities. We have done this by following 36,303 type 1 diabetes patients in the Swedish National Diabetes Register (NDR) for an average of 3.3 years. We mapped the incidence of cardiovascular disease, heart failure and chronic kidney disease (CKD), and evaluated the significance of these for premature death.

## Methods

All patients with type 1 diabetes listed in the Swedish National Diabetes Register (NDR) between January 1, 2015 and December 31, 2017 were included in this observational study^18^. Data regarding 36,303 patients were linked between NDR and other nationwide registers. The study was approved by the Swedish Ethical Review Authority. Individual consent is not required to report patients to national quality registries of healthcare, or to be included in a study like this, according to Swedish law (Patient Data Act 2008:355, chapter 7).

NDR, initiated 1996, contains information on the clinical characteristics, risk factors and complications of diabetes in patients aged 18 years or older, such as sex, age, diabetes duration, as well as levels of BMI, blood pressure, albuminuria, eGFR, HbA1c and blood lipids. Virtually all patients in Sweden with type 1 diabetes are included. In this study, we use an epidemiological definition of type 1 diabetes, i.e., people who are only treated with insulin and are 30 years or younger at the onset of diabetes.

Patient data were linked to several national health registers with complete coverage, through the unique social security number of everyone, to provide detailed information regarding baseline characteristics as well as outcomes. These links were implemented by Statistics Sweden, which returned anonymized data. The National Patient Register contains nationwide information on all hospitalizations and specialized outpatient visits from 1987^19^. Diagnoses are classified according to the International Classification of Diseases (ICD 9th and 10th Revision). Time and main or secondary causes of death have been recorded in the Cause of Death Register since 1961^20^. The Swedish Prescribed Drug Register (SPDR) contains detailed data on all prescriptions including ATC-codes filled at all pharmacies since 2005^21^. Demographic and socioeconomic data (from 1990 and onwards) was retrieved from LISA (Longitudinal Integration Database for Health Insurance and Labour Market studies), a database that holds annual registers of all Swedes since 1990^22^.

Normo-, micro-, and macroalbuminuria, as well as eGFR (G1-G5) and CKD staging (low-very high) was done in accordance with Kidney Disease: Improving Global Outcomes (KDIGO) guidelines (Supplementary Table S1a, S1b, S1c)^23^. The estimated glomerular filtration rate (eGFR) was calculated using the MDRD prediction equation^24^. History of CVD (including CHD, AMI, and stroke), heart failure, atrial fibrillation, and cancer were defined using ICD codes (Supplementary table S2). Mortality was assessed across categories of risk factors, CKD and for patients with a history of CVD or HF. The main outcome was time to mortality of any cause. All methods were performed in accordance with the relevant guidelines and regulations.

### Statistical analysis

We followed the patients from their index date (date of first registration in NDR from January 1, 2015) until December 31, 2018. The study approach expects virtually no dropout as the number of patients who emigrate is assumed to be insignificant, and data from the cause of death registry is complete. No patients were excluded during follow-up. Mortality rates were calculated as the number of events per 100 person years where person years are counted to censoring or death. The estimated rates are fitted with exact 95% confidence intervals. We used survival analysis with Kaplan–Meier plots and Cox regression analyses to assess the association between all available risk factors and mortality. Assessment of Schoenfeld’s residuals showed that the proportional hazard assumption was fulfilled. Standard errors were used to calculate the corresponding confidence intervals using the delta method, resulting in symmetric confidence intervals based on a normal approximation on the ratio scale. Missing data has not been imputed but only presented as raw data in the description of results and consequently the statistical modeling was done based on persons with complete data. The association between risk factors and outcome measures were considered statistically significant if *p* < 0.05, and confidence intervals (CI) were calculated to a confidence level of 95%. R statistical software (version 4.0.2; R Foundation for Statistical Computing, Vienna, Austria) was used for all calculations.

## Results

The baseline characteristics of the 36,303 people with type 1 diabetes included in the study according to age groups are given in Table 1. Men were in majority (55.2%), the mean age was 40.1 years, 73.2% were up to 20 years of age at the onset of diabetes, and the mean number of years with diabetes at the start of follow-up in this study was 25.5 years. The mean HbA1c was 64.1 mmol/mol (8.0%), blood pressure 125.6/73.2 mmHg, and LDL cholesterol 2.5 mmol/L. Information on marital status, country of birth, smoking and physical activity are given in Supplementary table S3.

**Table 1:** Baseline characteristics.

Age group (years)	18–49	50–59	60–69	≥ 70	Total
N	25,762	5121	3584	1836	36,303
% of total	71.0	14.1	9.9	5.1	
Age, mean (years, CI)	31.5 (31.4, 31.6)	54.1 (54.1, 54.2)	64.3 (64.2, 64.4)	74.7 (74.5, 74.9)	40.1 (40.0–40.3)
Age, median (range)	30.0 (18.0, 49.0)	54.0 (50.0, 59.0)	64.0 (60.0, 69.0)	73.0 (70.0, 96.0)	38.0 (18.0–96.0)
Sex (male, %)	56.0	54.3	52.1	53.1	55.2
**Age at onset of diabetes**					
0–10 years	9080 (35.2%)	1348 (26.3%)	784 (21.9%)	342 (18.6%)	11,554 (31.8%)
11–15 years	6621 (25.7%)	1179 (23.0%)	770 (21.5%)	366 (19.9%)	8936 (24.6%)
16–20 years	4197 (16.3%)	895 (17.5%)	647 (18.1%)	353 (19.2%)	6092 (16.8%)
21–25 years	3535 (13.7%)	877 (17.1%)	691 (19.3%)	382 (20.8%)	5485 (15.1%)
26–30 years	2329 (9.0%)	821 (16.0%)	692 (19.3%)	393 (21.4%)	4235 (11.7%)
Years with diabetes at index (years, CI)	18.0 (17.9, 18.2)	38.6 (38.4, 38.8)	47.6 (47.3, 47.9)	57.3 (56.9, 57.7)	25.8 (25.7, 26.0)
HbA1c (mmol/mol)	64.6 (64.4, 64.8)	64.3 (63.9, 64.7)	61.7 (61.3, 62.1)	60.7 (60.2, 61.2)	64.1 (63.9, 64.2)
HbA1c (%)	8.1 (8.0, 8.1)	8.0 (8.0, 8.1)	7.8 (7.8, 7.8)	7.7 (7.7, 7.8)	8.0 (8.0, 8.0)
BMI (kg/m2)	25.8 (25.7, 25.8)	26.7 (26.6, 26.8)	26.1 (25.9, 26.3)	25.5 (25.3, 25.7)	25.9 (25.9, 26.0)
Systolic blood pressure (mmHg)	122.3 (122.1, 122.5)	132.1 (131.6, 132.5)	134.7 (134.2, 135.2)	134.9 (134.2, 135.6)	125.6 (125.5, 125.8)
Diastolic blood pressure (mmHg)	74.0 (73.9, 74.1)	73.4 (73.1, 73.6)	69.9 (69.6, 70.2)	67.7 (67.3, 68.2)	73.2 (73.1, 73.3)
LDL cholesterol (mmol/L)	2.6 (2.6, 2.6)	2.5 (2.5, 2.6)	2.4 (2.4, 2.4)	2.3 (2.2, 2.3)	2.5 (2.5, 2.5)
HDL cholesterol (mmol/L)—Women	1.7 (1.7, 1.7)	1.9 (1.9, 1.9)	1.9 (1.9, 1.9)	1.8 (1.8, 1.9)	1.8 (1.7, 1.8)
HDL cholesterol (mmol/L)—Men	1.4 (1.4, 1.4)	1.6 (1.6, 1.6)	1.6 (1.6, 1.6)	1.6 (1.5, 1.6)	1.5 (1.5, 1.5)
Triglycerides (mmol/L)	1.1 (1.1, 1.1)	1.2 (1.1, 1.2)	1.1 (1.1, 1.1)	1.1 (1.1, 1.1)	1.1 (1.1, 1.1)
eGFR (mL/min/1.73m2)	103.7 (103.4, 104.1)	83.3 (82.6, 84.1)	76.2 (75.4, 77.1)	69.4 (68.3, 70.6)	95.9 (95.6, 96.2)
**eGFR stage**					
G1	15,167 (58.9%)	1793 (35.0%)	908 (25.3%)	298 (16.2%)	18,166 (50.0%)
G2	5171 (20.1%)	2084 (40.7%)	1596 (44.5%)	790 (43.0%)	9641 (26.6%)
G3a	312 (1.2%)	303 (5.9%)	341 (9.5%)	328 (17.9%)	1284 (3.5%)
G3b	147 (0.6%)	165 (3.2%)	208 (5.8%)	173 (9.4%)	693 (1.9%)
G4	104 (0.4%)	93 (1.8%)	111 (3.1%)	60 (3.3%)	368 (1.0%)
G5	84 (0.3%)	80 (1.6%)	55 (1.5%)	17 (0.9%)	236 (0.7%)
Missing	4777 (18.5%)	603 (11.8%)	365 (10.2%)	170 (9.3%)	5915 (16.3%)
**Albuminuria**					
Normoalbuminuria	18,577 (72.1%)	3457 (67.5%)	2285 (63.8%)	1076 (58.6%)	25,395 (70.0%)
Microalbuminuria	1397 (5.4%)	632 (12.3%)	578 (16.1%)	359 (19.6%)	2966 (8.2%)
Macroalbuminuria	671 (2.6%)	396 (7.7%)	306 (8.5%)	151 (8.2%)	1524 (4.2%)
Missing	5117 (19.9%)	636 (12.4%)	415 (11.6%)	250 (13.6%)	6418 (17.7%)
**CKD stage**					
Low	20,852 (80.9%)	3693 (72.1%)	2385 (66.5%)	1102 (60.0%)	28,032 (77.2%)
Moderate	1292 (5.0%)	503 (9.8%)	445 (12.4%)	232 (12.6%)	2472 (6.8%)
High	489 (1.9%)	286 (5.6%)	248 (6.9%)	223 (12.1%)	1246 (3.4%)
Very High	384 (1.5%)	343 (6.7%)	335 (9.3%)	196 (10.7%)	1258 (3.5%)
Missing	2745 (10.7%)	296 (5.8%)	171 (4.8%)	83 (4.5%)	3295 (9.1%)
History of CVD	428 (1.7%)	805 (15.7%)	1101 (30.7%)	839 (45.7%)	3173 (8.7%)
History of heart failure	120 (0.5%)	179 (3.5%)	299 (8.3%)	311 (16.9%)	909 (2.5%)
History of atrial fibrillation	91 (0.4%)	102 (2.0%)	205 (5.7%)	233 (12.7%)	631 (1.7%)
History of cancer	376 (1.5%)	313 (6.1%)	445 (12.4%)	352 (19.2%)	1486 (4.1%)
**Lipid lowering treatment**					
Yes	4414 (17.1%)	3249 (63.4%)	2662 (74.3%)	1373 (74.8%)	11,698 (32.2%)
No	19,148 (74.3%)	1658 (32.4%)	813 (22.7%)	415 (22.6%)	22,034 (60.7%)
Missing	2200 (8.5%)	214 (4.2%)	109 (3.0%)	48 (2.6%)	2571 (7.1%)
**Aspirin treatment**					
Yes	697 (2.7%)	1386 (27.1%)	1644 (45.9%)	1047 (57.0%)	4774 (13.2%)
No	22,602 (87.7%)	3394 (66.3%)	1762 (49.2%)	706 (38.5%)	28,464 (78.4%)
Missing	2463 (9.6%)	341 (6.7%)	178 (5.0%)	83 (4.5%)	3065 (8.4%)

Means, proportions and 95% confidence intervals (CI).

Kidney function, i.e., eGFR, eGFR stage, albuminuria and CKD stages are also given in Table 1. The overall mean eGFR was 95.9 mL/min/1.73 m^2^, and 70.0% were normoalbuminuric. Consequently, 77.2% of the patients were at a low CKD stage, but 6.8% presented moderate CKD, 3.4% high and 3.5% very high. A baseline record of previous CVD (including CHD, stroke, AMI) was found in 8.7%, heart failure in 2.5%, atrial fibrillation in 1.7%, and cancer in 4.1% of all patients; all increasingly prevalent in patients who were 60 years of age or older. Medical treatments according to risk groups are provided in Supplementary table S4. Renin-angiotensin system acting agents were used by 35.6% of all patients, but by more than 90% of patients with CKD or heart failure. Similar proportions of the patients with prior CVD or heart failure were using some platelet inhibitor, as well as lipid lowering treatment.

The mean follow-up time was 3.3 years (median 3.6 years, range 0–4.0 years) resulting in a total number of 119,800 patient-years of follow-up. Of the total 36,303 patients, 1127 patients died, resulting in a crude total mortality rate of 0.92% deaths/year observed. All-cause mortality rate per year was 0.22% in the age group 18–49 years, 1.2% in age group 50–59 years, 2.6% in age group 60–69 years, and 6·2% in the group of people 70 years of age or older. The median age of death was 75.3 (95% confidence interval, CI, 74.2, 76.4) years in men and 76.1 (95% CI 75.5, 77.2) years in women. Causes of death (according to death certificates) are listed in Supplementary Table S5 according to eGFR stages. Overall, diabetes was cited as the most common cause of death, but kidney disease, heart failure and cardiac arrest were also common.

Table 2 gives mortality rates (number of events per 100 person years counted until censoring or death), as well as unadjusted and adjusted risk according to risk factors and risk groups. The mortality rates were higher for people older than 60 years and especially for those older than 70 years, but quite similar in women and men, circa 31 per 100 person years. However, the mortality risk was 20% higher in men after multivariate analysis. The lowest adjusted mortality rates were noted in people with BMI 25.0–29.9, HbA1c 7.0–7.8%, diastolic blood pressure 60–69 mmHg and systolic blood pressure 130–139 mmHg. Significantly elevated adjusted mortality risks were observed in people with BMI lower than 25, HbA1c less than 7.0%, and the lowest blood pressure categories, as well as in people with HbA1c higher than 7.8%, and the highest blood pressure categories.

**Table 2:** Mortality rates and risk according to risk factors and risk group, unadjusted and adjusted (Cox regression).

Variable	Group	N	Mortality rate (number of events per 100 person years, 95 CI)	HR (univariate analysis; 95 CI)	HR (multivariate analysis; 95 CI)
Sex	Female	16,246	0.90 (0.82, 0.98)	– (Reference)	– (Reference)
	Male	20,057	0.97 (0.9, 1.05)	1.08 (0.96–1.22)	1.20 (1.06–1.36)
Age (years)	18–49	25,762	0.22 (0.19, 0.25)	–	–
	50–59	5121	1.22 (1.07, 1.39)	5.51 (4.53–6.71)	2.59 (2.04–3.29)
	60–69	3584	2.73 (2.45, 3.04)	12.32 (10.30–14.74)	3.97 (3.11–5.27)
	≥ 70	1836	6.98 (6.33, 7.69)	31.63 (26.59–37.62)	6.98 (5.14–9.48)
BMI (kg/m^2^)	< 18.5	485	2.24 (1.61, 3.06)	3.09 (2.16, 4.42)	3.96 (2.76, 5.70)
	18.5–24.9	13,504	0.85 (0.77, 0.94)	1.16 (1.00, 1.36)	1.23 (1.05, 1.44)
	25.0–29.9	10,719	0.73 (0.65, 0.82)	–	–
	30.0–34.9	3639	1.10 (0.93, 1.3)	1.51 (1.23, 1.85)	1.24 (1.00, 1.52)
	35.0–39.9	919	1.08 (0.77, 1.48)	1.48 (1.03, 2.12)	1.19 (0.83, 1.72)
	≥ 40	293	1.37 (0.8, 2.2)	1.87 (1.07, 3.27)	1.45 (0.82, 2.54)
	Missing	6744			
HbA1c (%)	< 7.0%	8408	0.92 (0.82, 1.04)	1.16 (0.97, 1.38)	1.23 (1.03, 1.48)
	7.0–7.8%	8692	0.8 (0.7, 0.9)	–	–
	7.8–8.8%	10,053	0.93 (0.83, 1.04)	1.17 (0.98, 1.38)	1.18 (1.00, 1.40)
	8.8–9.6%	4230	0.99 (0.84, 1.16)	1.25 (1.01, 1,54)	1.40 (1.13, 1.73)
	≥ 9.6%	3995	1.10 (0.94, 1.29)	1.39 (1.13, 1.72)	1.72 (1.39, 2.13)
	Missing	925			
Diastolic blood pressure (mmHg)	< 60	1211	2.78 (2.31, 3.33)	2.44 (1.96, 3.04)	1.10 (0.88, 1.38)
	60–69	7510	1.14 (1.02, 1.28)	–	–
	70–79	13,914	0.78 (0.71, 0.87)	0.69 (0.59, 0.80)	1.10 (0.94, 1.29)
	80–89	9377	0.65 (0.57, 0.74)	0.57 (0.48, 0.68)	1.08 (0.89, 1.30)
	90–99	1427	0.85 (0.63, 1.14)	0.75 (0.54, 1.04)	1.62 (1.14, 2.31)
	100–109	217	1.26 (0.67, 2.21)	1.11 (0.57, 2.16)	2.14 (1.07, 4.29)
	≥ 110	29	3.42 (1.24, 8.23)	3.02 (0.97, 9.42)	2.92 (0.91, 9.35)
	Missing	2618			
Systolic blood pressure (mmHg)	< 110	3047	0.77 (0.62, 0.96)	0.81 (0.63, 1.05)	1.82 (1.38, 2.38)
	110–119	6525	0.57 (0.48, 0.68)	0.60 (0.48, 0.75)	1.31 (1.05, 1.64)
	120–129	10,403	0.59 (0.52, 0.67)	0.62 (0.51, 0.74)	1.00 (0.83, 1.21)
	130–139	7611	0.96 (0.84, 1.08)		
	140–149	3755	1.47 (1.27, 1.69)	1.54 (1.27, 1.86)	1.02 (0.84, 1.23)
	150–159	1351	1.80 (1.45, 2.21)	1.88 (1.46, 2.42)	0.93 (0.72, 1.20)
	≥ 160	1036	3.07 (2.54, 3.69)	3.23 (2.57, 4.07)	1.19 (0.93, 1.53)
	Missing	2575			
History of CVD	No	33,130	0.51 (0.47, 0.56)	–	–
	Yes	3173	5.60 (5.15, 6.07)	10.91 (9.71–12.26)	1.91 (1.66–2.20)
History of heart failure	No	35,394	0.71 (0.67, 0.76)	–	–
	Yes	909	11.12 (9.91, 12.44)	15.72 (13.75–17.97)	2.06 (1.76–2.40)
Albuminuria stage	Normal	24,419	0.48 (0.43, 0.53)	–	–
	Mild	976	1.21 (0.9, 1.61)	2.53 (1.84–3.50)	1.26 (0.91–1.74)
	Microalbuminuria	2966	2.39 (2.1, 2.7])	5.00 (4.25–5.87)	1.76 (1.48–2.09)
	Macroalbuminuria	1524	5.14 (4.54, 5.79)	10.80 (9.22–12.65)	2.35 (1.92–2.87)
	Missing	6418			
eGFR stage	G1	18,166	0.38 (0.33, 0.43)	–	–
	G2	9641	0.87 (0.77, 0.97)	2.28 (1.92–2.71)	0.90 (0.75–1.09)
	G3	1977	4.40 (3.92, 4.93)	11.62 (9.77–13.83)	1.47 (1.20–1.80)
	G4	368	9.11 (7.51, 10.98)	24.22 (19.17–30.60)	2.71 (2.06–3.57)
	G5	236	11.42 (9.11, 14.14)	30.53 (23.53–39.63)	3.81 (2.81–5.17)
	Missing	5915			

*Denotes the reference group.

Mortality was higher for people with a history of cardiovascular disease (37.2 per 100 person years) and especially for those with a history of heart failure (46.2 per 100 person years), which in both cases can be translated into an almost doubling of the risk for death after multivariate analyzes. Similarly, mortality risk increased in people with progressively more pronounced kidney disease, with 36.4 (per 100 person years) in macroalbuminuria and eGFR stage G3 35.3 (per 100 person years). Mortality risks were higher than 40 (per 100 person years) in G4 and G5, with hazard ratio 2.71 and 3.81 in relation to G1, respectively (Table 2).

**Figure 1:**
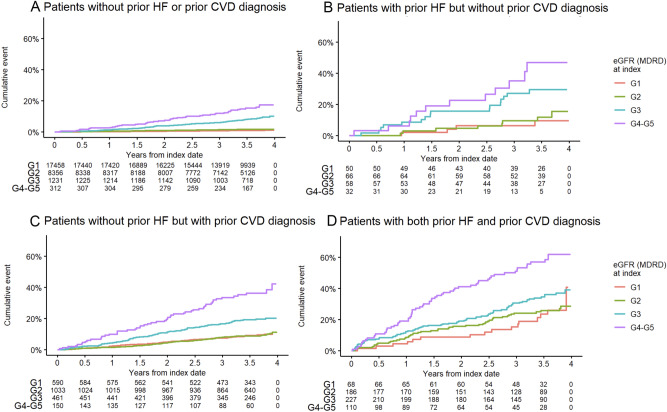
(**A**)–(**D**) Mortality according to previous history of CVD, heart failure and eGFR stages.

Figure 1A–D and Table 3 give mortality rates and risk of mortality adjusted for age at the start of follow-up in people with type 1 diabetes grouped according to history of CVD, heart failure and kidney function (eGFR stages). The highest mortality rates were seen in people with lowest kidney function (eGFR stages G4-G5), with previous CVD but particularly in people with previous heart failure. Any combination of these conditions was associated with elevated risk, but the risk estimates were more than two-fold higher in people with G4 or lower kidney function, irrespective of previous history of CVD or heart failure, or not.

**Table 3:** Mortality rates and risk according to risk group, adjusted for age at baseline (Cox regression).

History of CVD	History of heart failure	eGFR stage	N	Mortality rate (number of events per 1000 person years, 95 CI)	HR (univariate analysis)
No	No	G1	17,458	30.19 (29.74, 30,63)	–
		G2	8356	29.55 (28.93, 30.18)	0.77 (0.61, 0.97)
		G3	1231	32.30 (30.61, 34.06)	2.16 (1.64, 2.83)
		G4	198	34.75 (30.50, 39.45)	4.51 (2.95, 6.88)
		G5	114	37.20 (31.44, 43.73)	9.63 (6.1, 15.22)
		Missing	5535		
No	Yes	G1	50	33.53 (25.72, 43.04)	–
		G2	66	34.17 (27.24, 42.38)	1.05 (0.31, 3.53)
		G3	58	43.02 (34.29, 53.36)	2.3 (0.74, 7.16)
		G4	17	56.11 (38.12, 80.15)	5.65 (1.69, 18.92)
		G5	15	53.99 (34.72, 80.84)	4.75 (1.18, 19.09)
		Missing	32		
Yes	No	G1	590	32.74 (30.31, 35.32)	–
		G2	1033	32.20 (30.38, 34.11)	0.81 (0.58, 1.14)
		G3	461	37.35 (34.36, 40.54)	1.57 (1.11, 2.21)
		G4	87	44.44 (37.1, 52.85)	3.78 (2.42, 5.91)
		G5	63	53.22 (43.17, 64.97)	6.07 (3.75, 9.83)
		Missing	268		
Yes	Yes	G1	68	40.09 (32.41, 49.11)	–
		G2	186	41.79 (36.78, 47.32)	0.96 (0.55, 1.7)
		G3	227	47.25 (42.25, 52.69)	1.29 (0.75, 2.22)
		G4	66	66.13 (54.55, 79.49)	3.04 (1.69, 5.47)
		G5	44	73.39 (58.13, 91.55)	3.96 (2.12, 7.39)
		Missing	80		

## Discussion

In this nationwide study, we followed a large cohort with type 1 diabetes to describe the significance of risk factors and co-morbidities, such as CVD, heart failure, and CKD, regarding the risk of mortality. Elevated risk was demonstrated in older age groups and in men compared to women, but only very high HbA1c and blood pressure levels were statistically significant in multivariate analyzes, as well as underweight, or normal weight (but not overweight or obesity). A history of CVD or heart failure was found to be harmful, and to an even greater extent albuminuria and advanced stages of CKD. Each combination of these conditions further increased the risk of mortality, emphasizing the clinical significance of them and advocating optimized treatment and follow-up in the clinical practice of these individuals.

Long-term fluctuating glucose levels due to pancreatic beta cell destruction is the unique factor in type 1 diabetes that causes diabetic and particularly microvascular complications, in combination with other pathophysiological mechanisms^25,26^. The associations between hyperglycemia, concomitant risk factors, and CVD, heart failure, as well as CKD, have also long been established^7,27–29^. Optimized glucose control however reduces the risk of diabetic nephropathy and CVD, and has been suggested to increase life expectancy^6,30^, although multimodal treatment of risk factors is likely to be paramount^2,28^. Diabetic kidney disease is, in turn, strongly associated with CVD, heart failure and mortality through several mechanisms^31–33^. It has been previously reported that the risk of hospitalization for heart failure is doubled at eGFR 45–60 (mL/min/1.73m^2^) and more than three times higher at eGFR at < 30 (mL/min/1.73 m^2^) in type 1 diabetes patients^34^. The importance of diagnosis and adequate treatment of CKD in diabetes has been described in detail^35,36^. Our study provides further support for these relationships and the consequences also regarding the risk of mortality.

The relatively short follow-up time after baseline could reduce the impact of traditional risk factors in the long term, as suggested by the relatively low hazard for mortality depending on elevated levels of HbA1c and blood pressure. Interestingly, the lowest risk estimates were not noted in the groups with the lowest levels of these or BMI. However, the current results are consistent with findings in other studies and cohorts and may be caused by residual confounding or reverse causality^28,37,38^.

We noted a slightly elevated risk of death in men compared with women after multivariate analysis. Sex differences in cardiovascular outcomes, cancer and death in diabetes have recently been subject to a systematic review and meta-analysis, concluding that women generally are at higher risk, although there was a significant heterogeneity between studies and different parts of the world^39^. However, the current results seem to be broadly in line with previous results in Sweden, which have suggested a shorter expected survival for men with type 1 diabetes, but the reasons for this remain to be clarified^2^.

A strength of this study is the large and unselected sample of individuals with type 1 diabetes. Virtually all patients with type 1 diabetes in the country are reported annually to NDR. The study, which used detailed information on clinical characteristics, concomitant conditions, and medications, as well as socio-economic factors, is contemporary and addresses the current consequences of traditional risk factors and advanced diabetes complications in clinical practice. In observational studies, however, there is always a possibility of residual confounding, even if we in this study have taken known co-morbidities, clinical and laboratory characteristics as well as socioeconomic status into account. We did not include blood lipids, as annual measurements of these are not mandatory in young patients at low risk, in contrast to HbA1c and blood pressure; or patients diagnosed at higher age than 30 years, to avoid a more uncertain diagnosis of diabetes type. We did not compare with mortality in a cohort without diabetes, as this was done quite recently in a similar study, which showed an approximately threefold increase in mortality risk^1^. We can also only study individuals who were alive at the start of the current follow-up period, and not earlier or later cohorts with or without co-morbidities. Results might also appear different in other countries and cohorts.

The results of this nationwide study with 36,303 people with type 1 diabetes in a high-endemic country, which was designed to provide a contemporary view of the risk of mortality in type 1 diabetes at different levels of risk factors and in groups of people with already established cardiovascular and renal diabetes complications, emphasizes the clinical importance of these. In particular, people with a combination of chronic kidney or cardiovascular disease, and heart failure, exhibit a very markedly increased risk of dying during the follow-up time. These findings thus constitute strong arguments for optimized and individualized treatment of these groups of people with type 1 diabetes in clinical everyday life.

## Supplementary Information

Supplementary Information.

## Data Availability

The datasets generated during and/or analyzed during the current study are available from the corresponding author on reasonable request.
