# Supplementary material for: The significance of chronic kidney disease, heart failure and cardiovascular disease for mortality in type 1 diabetes: nationwide observational study
Source: Sci Rep. 2022 Oct 26;12:17950. doi: 10.1038/s41598-022-22932-4 (PMC9606313; doi:10.1038/s41598-022-22932-4)
Supplement: Supplementary file 1 — Supplementary Information. [file 41598_2022_22932_MOESM1_ESM.docx]

Supplementary Table 1a

Definition of eGFR staging

| eGFR stage | eGFR (mL/min/1.73 m2) | Category (loss of kidney function) |
| --- | --- | --- |
| G1 | ≥90 | Normal |
| G2 | 60-89 | Mild |
| G3a | 45-59 | Mild to moderate |
| G3b | 30-44 | Moderat to severe |
| G4 | 15-29 | Severe |
| G5 | <15 | Kidney failure |

Supplementary Table 1b

Definition of Albuminuria staging

| Albuminuria stage |  | Albumin/creatinine (mg/g) | Category |
| --- | --- | --- | --- |
| A1 | Normal | <10 | Normal |
| A1 | Mild | 10-29 | Mild |
| A2 | Microalbuminuria | 200-299 | Moderate |
| A3 | Macroalbuminuria | ≥300 | Severe |

Supplementary Table 1c

Definition of CKD (chronic kidney disease) staging

| CKD stage | Albuminuria stage | | |
| --- | --- | --- | --- |
| eGFR stage | A1 | A2 | A3 |
| G1 | Low | Moderate | High |
| G2 | Low | Moderate | High |
| G3a | Moderate | High | Very high |
| G3b | High | Very high | Very high |
| G4 | Very high | Very high | Very high |
| G5 | Very high | Very high | Very high |

Supplementary Table 2

Previous conditions according to ICD codes

| Characteristic | ICD9- or  ICD10-codes |
| --- | --- |
| History of CVD | 410, 431, 432, 433, 434, 436, I21, I61, I62, I63, I64 |
| History of atrial fibrillation | 427D, I48 |
| History of heart failure | 428, I50 |
| History of cancer | 140-208, C0-C9 |

Supplementary Table 3

Marital status, country of birth, smoking and physical activity

| Age group (years) | 18-49 | 50-59 | 60-69 | ≥70 | Total |
| --- | --- | --- | --- | --- | --- |
| Marital status |  |  |  |  |  |
| Married | 6412 (25.0%) | 2446 (48.1%) | 2081 (59.0%) | 1058 (59.9%) | 11997 (33.3%) |
| Separated | 1383 (5.4%) | 1004 (19.8%) | 676 (19.2%) | 271 (15.3%) | 3334 (9.2%) |
| Single | 17848 (69.5%) | 1555 (30.6%) | 621 (17.6%) | 161 (9.1%) | 20185 (56.0%) |
| Widowed | 31 (0.1%) | 76 (1.5%) | 149 (4.2%) | 277 (15.7%) | 533 (1.5%) |
| Country of birth |  |  |  |  |  |
| Africa | 334 (1.3%) | 33 (0.6%) | 10 (0.3%) | 1 (0.1%) | 378 (1.0%) |
| Asia | 695 (2.7%) | 77 (1.5%) | 20 (0.6%) | 7 (0.4%) | 799 (2.2%) |
| EU28 (Nordic excluded) | 421 (1.6%) | 42 (0.8%) | 22 (0.6%) | 20 (1.1%) | 505 (1.4%) |
| Nordic (Sweden excluded) | 320 (1.2%) | 181 (3.5%) | 118 (3.3%) | 52 (2.8%) | 671 (1.8%) |
| Sweden | 23607 (91.6%) | 4723 (92.2%) | 3386 (94.5%) | 1746 (95.1%) | 33462 (92.2%) |
| Smoking - Women |  |  |  |  |  |
| Never | 7707 (68.0%) | 1364 (58.3%) | 1001 (58.3%) | 562 (65.3%) | 10634 (65.5%) |
| Previous | 953 (8.4%) | 419 (17.9%) | 365 (21.3%) | 136 (15.8%) | 1873 (11.5%) |
| Occasional | 281 (2.5%) | 38 (1.6%) | 16 (0.9%) | 7 (0.8%) | 342 (2.1%) |
| Daily | 983 (8.7%) | 268 (11.5%) | 131 (7.6%) | 30 (3.5%) | 1412 (8.7%) |
| Missing | 1407 (12.4%) | 249 (10.7%) | 203 (11.8%) | 126 (14.6%) | 1985 (12.2%) |
| Smoking - Men |  |  |  |  |  |
| Never | 9972 (69.1%) | 1745 (62.7%) | 1011 (54.1%) | 561 (57.5%) | 13289 (66.3%) |
| Previous | 1156 (8.0%) | 455 (16.3%) | 467 (25.0%) | 232 (23.8%) | 2310 (11.5%) |
| Occational | 434 (3.0%) | 50 (1.8%) | 25 (1.3%) | 5 (0.5%) | 514 (2.6%) |
| Daily | 1021 (7.1%) | 200 (7.2%) | 129 (6.9%) | 24 (2.5%) | 1374 (6.9%) |
| Missing | 1848 (12.8%) | 333 (12.0%) | 236 (12.6%) | 153 (15.7%) | 2570 (12.8%) |
| Physical activity - Women (per week) |  |  |  |  |  |
| Never | 643 (5.7%) | 203 (8.7%) | 169 (9.8%) | 132 (15.3%) | 1147 (7.1%) |
| <1 | 1344 (11.9%) | 276 (11.8%) | 215 (12.5%) | 116 (13.5%) | 1951 (12.0%) |
| 1-2 | 2430 (21.4%) | 473 (20.2%) | 323 (18.8%) | 162 (18.8%) | 3388 (20.9%) |
| 3-5 | 3099 (27.3%) | 543 (23.2%) | 359 (20.9%) | 156 (18.1%) | 4157 (25.6%) |
| 5-7 | 2064 (18.2%) | 519 (22.2%) | 437 (25.5%) | 179 (20.8%) | 3199 (19.7%) |
| Missing | 1751 (15.5%) | 324 (13.9%) | 213 (12.4%) | 116 (13.5%) | 2404 (14.8%) |
| Physical activity - Men (per week) |  |  |  |  |  |
| Never | 1012 (7.0%) | 247 (8.9%) | 199 (10.7%) | 145 (14.9%) | 1603 (8.0%) |
| <1 | 1873 (13.0%) | 368 (13.2%) | 245 (13.1%) | 130 (13.3%) | 2616 (13.0%) |
| 1-2 | 2995 (20.8%) | 550 (19.8%) | 343 (18.4%) | 143 (14.7%) | 4031 (20.1%) |
| 3-5 | 3999 (27.7%) | 710 (25.5%) | 379 (20.3%) | 147 (15.1%) | 5235 (26.1%) |
| 5-7 | 2501 (17.3%) | 563 (20.2%) | 484 (25.9%) | 277 (28.4%) | 3825 (19.1%) |
| Missing | 2051 (14.2%) | 345 (12.4%) | 218 (11.7%) | 133 (13.6%) | 2747 (13.7%) |

Numbers and proportions

Supplementary Table 4

Medical treatments according to risk groups

|  | Patient group | | | | | | |
| --- | --- | --- | --- | --- | --- | --- | --- |
|  | CKD low | CKD medium/high | No history of CVD | History of CVD | No history of heart failure | History of heart failure | Total |
| N | 27807 | 2581 | 33130 | 3173 | 35394 | 909 | 36303 |
| Lipid lowering treatment | 10928 (39.5%) | 2228 (86.4%) | 11841 (35.9%) | 2988 (94.2%) | 14011 (39.8%) | 818 (90.2%) | 14829 (41.1%) |
| *Antihypertensive* | *361 (1.3%)* | *491 (19.0%)* | *613 (1.9%)* | *347 (10.9%)* | *801 (2.3%)* | *159 (17.5%)* | *960 (2.7%)* |
| RAS acting agents | 8980 (32.4%) | 2357 (91.4%) | 10013 (30.4%) | 2833 (89.3%) | 11985 (34.0%) | 861 (94.9%) | 12846 (35.6%) |
| Loop diuretics | 2155 (7.8%) | 1709 (66.2%) | 2653 (8.1%) | 1688 (53.2%) | 3550 (10.1%) | 791 (87.2%) | 4341 (12.0%) |
| Calcium channel blockers | 3612 (13.0%) | 1730 (67.1%) | 4266 (13.0%) | 1746 (55.0%) | 5450 (15.5%) | 562 (62.0%) | 6012 (16.6%) |
| Beta blockers | 4241 (15.3%) | 1702 (66.0%) | 4199 (12.7%) | 2556 (80.6%) | 5931 (16.8%) | 824 (90.8%) | 6755 (18.7%) |
| Aspirin | 4647 (16.8%) | 1635 (63.4%) | 4200 (12.7%) | 2841 (89.6%) | 6277 (17.8%) | 764 (84.2%) | 7041 (19.5%) |
| Platelet inhibitors | 6035 (21.8%) | 1811 (70.2%) | 5863 (17.8%) | 3013 (95.0%) | 8056 (22.9%) | 820 (90.4%) | 8876 (24.6%) |
| Digoxin | 49 (0.2%) | 48 (1.9%) | 41 (0.1%) | 68 (2.1%) | 39 (0.1%) | 70 (7.7%) | 109 (0.3%) |
| Nitrates | 1361 (4.9%) | 679 (26.3%) | 409 (1.2%) | 1881 (59.3%) | 1810 (5.1%) | 480 (52.9%) | 2290 (6.3%) |

Numbers and proportions

Supplementary Table 5

Causes of death according to eGFR stages

|  | Patient group | | | | | |
| --- | --- | --- | --- | --- | --- | --- |
|  | G1 (n=230) | G2 (n = 286) | G3 (n=282) | G4-G5 (n=176) | Missing G stratum (n=153) | Total (n=1127) |
| Cancer | 39 (17%) | 56 (19.6%) | 27 (9.6%) | 12 (7.4%) | 24 (15.7%) | 159 (14.1%) |
| Diabetes | 141 (61.3%) | 161 (56.3%) | 187 (66.3%) | 134 (76.1%) | 89 (58.2%) | 712 (63.2%) |
| Cardiac arrest | 31 (13.5%) | 49 (17.1%) | 54 (19.1%) | 32 (18.2%) | 28 (18.3%) | 194 (17.2%) |
| Heart failure | 13 (5.7%) | 50 (17.5%) | 89 (31.6%) | 42 (23.9%) | 33 (21.6%) | 227 (20.1%) |
| Kidney disease | (2.2%) | 22 (7.7%) | 83 (29.4%) | 122 (69.3%) | 35 (22.9%) | 267 (23.7%) |
| CVD | 19 (8.3%) | 25 (8.7%) | 23 (8.2%) | 13 (7.4%) | 10 (6.5%) | 90 (8.0%) |

Numbers and proportions
